# Protective and therapeutic effectiveness of taurine supplementation plus low calorie diet on metabolic parameters and endothelial markers in patients with diabetes mellitus: a randomized, clinical trial

**DOI:** 10.1186/s12986-022-00684-2

**Published:** 2022-07-23

**Authors:** Jalal Moludi, Shaimaa A. Qaisar, Mustafa M. Kadhim, Yasin Ahmadi, Mina Davari

**Affiliations:** 1 School of Nutritional Sciences and Food Technology, Kermanshah University of Medical Sciences, Kermanshah, 5166614711 Iran; 2 Behavioral Disease Research Center, Kermanshah University of Medical Sciences, Kermanshah, Iran; 3 Chemistry Department, College of Education, University of Garmian, Sulimmania, Iraq; 4 Department of Medical Laboratory Techniques, Dijlah University College, Baghdad, 10021 Iraq; 5 Medical Laboratory Techniques Department, Al-Farahidi University, Baghdad, Iraq; 6 Tabriz University of Medical Sciences, Kermanshah, Iran

**Keywords:** Diabetes mellitus, Endothelial markers, Glycemic control, Inflammation, Taurine

## Abstract

**Background:**

Taurine supplementation as a sulfur-containing amino acid may attenuate and/or alleviate diabetes-induced complications and endothelial dysfunction via its anti-inflammatory and antioxidant activities. Our purpose was to investigate the effect of Taurine supplementation on endothelial dysfunction markers, oxidative stress, inflammation, and glycemic control in patients with type 2 diabetes mellitus (T2DM).

**Methods:**

In the current clinical trial, 120 patients with T2DM were randomly allocated to take either Taurine (containing 1 g Taurine, n = 60) or placebo (n = 60) three times per day for an eight-week period. Moreover, all patients were on a low-calorie diet. The primary outcome was fasting blood glucose (FBG) and endothelial markers including sera intercellular adhesion molecule 1 (ICAM-1), vascular cell adhesion molecule (VCAM), and matrix metallopeptidase 9 (MMP-9). The secondary outcome was dietary intake, anthropometric indices, serum insulin and Homeostatic Model Assessment of Insulin Resistance (HOMA-IR), total antioxidant capacity (TAC), tumor necrosis factor (TNF), high-sensitivity C-reactive protein (hs-CRP), malondialdehyde (MDA), and lipid profile.

**Results:**

After 8 weeks, Taurine-supplemented patients had a considerable decrease in serum insulin and HOMA-IR compared to placebo group. However, Taurine supplementation did not improve other metabolic parameters including lipid profiles, glycated hemoglobin, and fasting blood glucose (FBG). There was a significant decline in MDA, TNF, and hs-CRP levels after these eight-week period of Taurine supplementation. In addition, the Taurine group had fewer serum levels of endothelial dysfunction markers than the placebo group.

**Conclusions:**

The evidence from our study revealed that Taurine supplementation significantly reduced insulin and HOMA-IR, as well as oxidative stress, inflammation, and endothelial markers in individuals with T2DM.

*Trial registration* The protocol of the study was recorded in the Iranian Registry of Clinical Trials (IRCT20180712040438N3).

## Introduction

Type 2 diabetes mellitus (T2DM) has a significant social and economic burden which is identified by high blood glucose levels (hyperglycemia) due to insulin resistance, insulin secretion, or both [1]. The persistent hyperglycemia results in extended damage including endothelial markers, oxidative stress, and is associated with complications such as nephropathy, neuropathy, cardiovascular disease, retinopathy, etc. [2, 3]. Individuals with T2DM are at a higher predictive risk of peripheral vascular, cardiovascular, and cerebrovascular disease [4]. However, the underlining mechanisms of these complications are not well understood. One possible link is endothelial markers, which have implicated in these conditions [5, 6]. Endothelial markers are described by a change in the activities of the endothelium towards reduced vasodilation, a proinflammatory state, and prothrombic properties [7]. In diabetes, many mechanisms including insulin resistance and oxidative stress may trigger endothelial dysfunction [8–10].

Taurine (2-aminoethanesulfonic acid) is also an amino acid found in substantial amounts in mammalian tissues and can act as an antioxidant [11]. Although biosynthesis is also strictly dependent upon the cysteine in the existence of cysteine dioxygenase, Taurine is mostly attained from dietary intakes, for instance, meat, seafood, and eggs [12]. Epidemiological investigations have confirmed a decline in plasma Taurine in diabetic subjects [13]. Some studies have stated that Taurine supplementation can reduce cardiovascular risk (CV) factors [14, 15]. Taurine has been studied in doses of 1 to 6 g/day (treatment duration range, 1 to 8 weeks) in a previous clinical trials [16, 17]. Taurine is one of the keys component of glucose homeostasis since it has insulin-like actions and prohibits ATP-sensitive K^+^ channels thereby playing a crucial role in insulin secretion [18]. Interestingly, Taurine ameliorates the Uncoupling protein (UCP)-2 overexpression in *β*-cells of the pancreas [19]. Notably, Taurine scavenges hypochlorous acid formed from granulocytes activation [20]. Furthermore, it may act more as an indirect antioxidant [21].

A limited number of intervention studies examined the effects of taurine administration on biomarkers of endothelial dysfunction and inflammation. Taurine administration attenuates endothelial and cardiac dysfunction in male Sprague–Dawley rats [22]. Likewise, taurine supplementation had a beneficial impact on macrovascular function, assessed by FMD, young male type 1 diabetics [23]. Up to now earlier, human intervention studies have only investigated the effects of taurine on a limited number of biomarkers of endothelial dysfunction. So, it seems worthwhile to unravel the role of Taurine supplementation on endothelial cells in patients with T2DM.. to the best of our knowledge, there are a limited number of clinical trials evaluating the impact of Taurine supplementation on endothelial dysfunction in patients with T2DM. Considering the anti-oxidant [21], insulin-sensitizing actions [24], hypoglycemic properties [25], and pro-lipolytic effects of Taurine [19], our study aimed to evaluate the impact of Taurine supplementation along with calorie restriction on endothelial, oxidative stress, and inflammation markers, as well as glycemic control in patients with T2DM.

## Methods

### Participants

This randomized, double-blind, clinical trial was carried out in Kermanshah, Iran, in accordance with the Helsinki Declaration. The proposal was approved by research ethics committee of the Kermanshah University of Medical Sciences (IR.KUMS.REC.1398.1187). Also, the protocol of the study was recorded in the Iranian Registry of Clinical Trials (IRCT20180712040438N3). Written informed consent was *achieved* from all *participants* whereby the benefits and risks of the study were clarified. The main outcomes were the alterations from baseline in FBG and markers of endothelium dysfunction. The secondary endpoints were the changes in lipid profile (LDL-C, HDL-C, total cholesterol and, triglycerides), glycemic control (HbA1c, insulin), hs-CRP, and serum total antioxidant capacity (TAC) and malondialdehyde (MDA). A sample size of at least 60 participants per group was calculated using the standard formula and as type I error (alpha) = 0.05, study power = 0.80, and assuming at least a 5% decrease in FBG levels post-intervention, the required sample size was calculated as 120 cases.

**Fig. 1:**
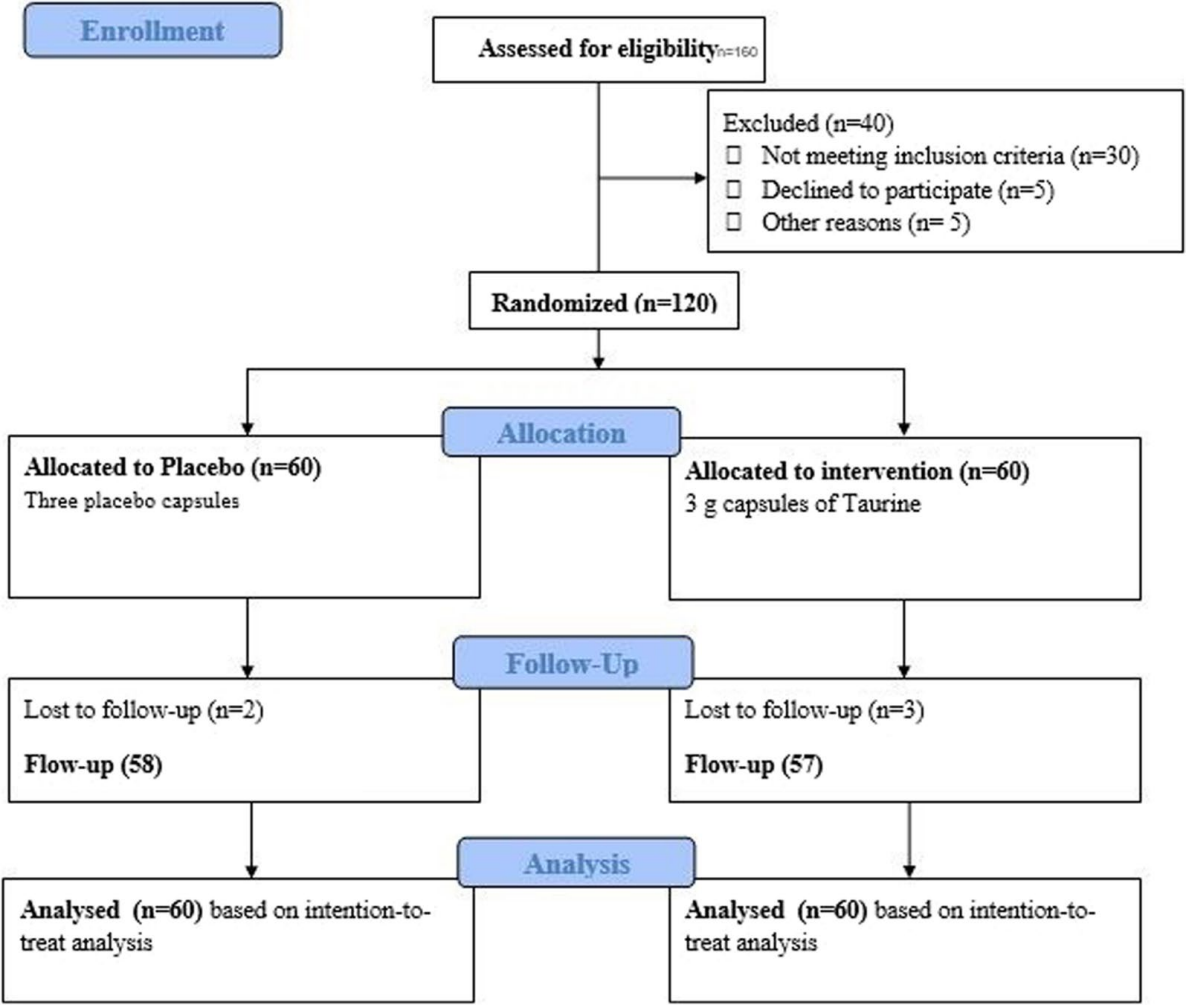
Flowchart of study

All individuals were screened by a physician for eligibility. For this study, 120 patients aged 30–60, of both genders, and with a body mass index (BMI) of 25–35 kg/m^2^ were enrolled (see flowchart in Fig. 1). Individuals were excluded if they had glycated hemoglobin (HbA1c) above 11.0%, used insulin, corticosteroid, or non-steroidal anti-inflammatory drugs; if they had renal, thyroid, pancreatic, unstable angina or stroke, or liver diseases; if they were pregnant or breastfeeding; if they are consuming vitamins/minerals for no less than 3 months before the trial. Also, patients with severe diabetes complications (including proliferative diabetic retinopathy, microalbuminuria, and peripheral neuropathy) were excluded. All participants had received stable treatment with oral glucose-lowering agents.

A third party who was unaware of the investigation provided the allocation sequence taken out from Random Allocation Software (RAS). The patients, researchers, and the medical suppliers were blinded after assignment to trial. After the screening of 280 patients with T2DM, a whole number of 160 subjects were eligible for this investigation. Amongst them, 120 patients consented to be randomized to receive either Taurine or placebo daily for an eight-week duration (Fig. 1). A total of 115 participants completed the study, and no individual in either group reported any side effects and tolerated the supplement very well. Based on the intention-to-treat (ITT) principle, we analyzed all participants (n = 120) in the final analysis.

### Intervention and dietary plan

Participants who fulfilled all suitability criteria were randomly allocated to one of two groups: 1000 mg Taurine (n = 60) (Karen Food Supplement Co, > 98% pure, Tehran, Iran), or 1000 mg starch as placebo (n = 60) three times per day for 8 weeks. The quality placebo of the placebo was alike Taurine in terms of shape, size, and packaging. Weekly contacts were applying to confirm obedience. Compliance with supplementation was established by requesting participants to return the medication containers. Participants with more than 10% unused capsules were excluded from the study.

All patients were on a moderately calorie-restricted diet plan, with a 500 kcal/day deficit compared to the total energy expenditure (TEE). The regime was planned to facilitate 5–7.5% of weight loss, at a rate of 0.5 kg/week. Calorie intake was planned based on individual features of the participants and with the aim of daily energy restriction (500 kcal fewer than the TEE. We assessed baseline energy requirements using the Mifflin formula and multiplied baseline energy requirements with physical activity level [14, 26] estimated according to self-reported physical activity to calculate the TEE. TEE is comprised of Resting Energy Expenditure (REE), Activity Energy Expenditure (AEE), and Thermic Effect of Food (TEF). In the low-calorie group, dirt was provided 10 ~ 15% from protein, 55 ~ 60% of TEE from carbohydrate, and 25 ~ 35% from fat. Meal plans were arranged based on these calculations, and according to the food-based dietary guidelines for Iranians. The composition of the study diet included the following daily servings: 7–9 servings of whole grains; 2–4 servings of fruits; 2–4 servings of vegetables; 2–3 servings of dairy (low-fat/ non-fat); 3–4 servings of lean meat; 3 servings/week of legumes; 1 serving of sweets.

### Dietary assessment

Dietary intake was measured by a dietary record at weeks 0, 4, and 8 of the intervention. We applied Nutritionist IV software (First Databank Division, the Hearst Corporation, San Bruno, CA, USA) in sync for Iranian diets to obtain nutrient intakes of participants [26–28].

## Physical activity levels

The International Physical Activity Questionnaire Short-Form (IPAQ-SF) which is a self-report questionnaire were used to measure physical activity.

### Assessment of anthropometric indices

Bodyweight was measured via a scale with 0.250 g precision (Seca, Germany) and participants were measured while wearing the light cloths and without shoes. Height without shoes was assessed by a tape with 1-cm precision. BMI was calculated by dividing weight (Kg) by height^2^ (m). To reduce measurement bias, all data was measured by the same trained dietitians.

All participants were examined for systolic and diastolic blood pressure (SBP, DBP, respectively) through calibrated tools.

### Biochemical variables

After 8–12 h of fasting, blood was taken from participants and stored until analyses. The serum lipid levels including, High-density lipoprotein cholesterol (HDL-C), total cholesterol [6], and triglyceride (TG) levels were measured by enzymatic kits (Pars Azmun, Iran). Friedewald formula was applied to compute Low-density lipoprotein cholesterol (LDL-C) levels. Serum Fasting Blood Sugar (FBG) level was assessed by the glucose oxidase method by an available kit (Pars Azmun, Iran). Commercial kits were used to measure insulin (Pars Azmun, Karaj, Iran) and HbA1c levels (Pishtaz Teb, Iran). HOMA-IR was computed using formula: [(fasting insulin (mU/mL) × FBG (mg/dL)]/405).

### Biomarker of inflammation and oxidative stress

The level of high sensitivity C-reactive protein (hs-CRP) was measured through immunoturbidimetry. The serum concentration of malondialdehyde (MDA) was assayed according to the reaction of MDA with thiobarbituric acid. The serum total antioxidant capacity (TAC) was measured using commercial kits (Glory Science Co.). Serum levels of Tumor Necrosis Factor (TNF) were assessed through an enzyme-linked immunosorbent assay (ELISA) (Crystal Day, Shanghai) as stated by the manufacturer’s instructions.

### Biomarkers of endothelial markers.

Sera level of intercellular adhesion molecule 1 (ICAM-1), vascular cell adhesion molecule (VCAM), and matrix metallopeptidase 9 (MMP-9) concentrations were determined by enzyme-linked immunosorbent assay using commercial kits (Shanghai Crystal Day Biotech Co., Ltd.).

### Statistical analysis

SPSS software (version 16; SPSS Inc., Chicago, IL) was used to analyses data, and data were reported as mean and standard deviation (SD). To decide the normality of data, we applied skewness and kurtosis test. We used paired samples t-test statistics to report within-group comparisons. for adjusting group comparisons for differences in the distribution of confounders, we did an analysis of covariance (ANCOVA). To avoid the effects of dropout, the ITT approach was conducted [29]. In order to obtain statistical representative, a *P* value of less than 0.05 was considered statistically significant.

## Results

Two patients in the placebo group were excluded because insulin therapy (n = 1) or hospitalization (n = 1), and 3 patients in the taurine group were also excluded because of insulin therapy (n = 2) or physical illness (n = 1). To end with, based on the ITT principle, we included all participants (n = 120) in the analyzes. Figure 1 is presented the study *Flowchart*. Baseline medical and bassline and general characteristics of the study participants are set out in Table.1. No statically significant differences were observed between the two groups in terms of sex ratio, weight, BMI, duration of disease in years (taurine group: 5.10 ± 1.30; and placebo group: 4.9 ± 1.20, p = 0.407), smoking history, physical activity levels and % of patients using glucose-lowering agents/drugs (*P* > 0.050). The mean age was 52.55 (± 8.5) years. A total of 65% of the participants had hypertension, and 30% had hypercholesterolemia.

**Table 1:** General characteristics of the study participants

Variable	Taurine group (n = 60)	Placebo group (n = 60)	*P*-value
Age (years)^a^	52.13 ± 8.1	53.08.1 ± 8.8	0.686^d^
Gender [17]^b^	48 (80)	49 (81)	0.500
Weight^a^ (kg) at study baseline	78.88 ± 8.3	79.2 ± 9.1	0.835^d^
Weight (kg) after intervention	75.44 ± 9.4	77.10 ± 8.2	0.315
BMI at study baseline	27.94 ± 6.9	28.01 ± 7.1	0.835^d^
BMI after intervention	26.80 ± 9.4	27.25 ± 8.2	0.225
Diabetes duration (years)	5.1 ± 1.3	4.9 ± 1.2	0.407
Active Smoking^b^	5 (8)	4 (6)	0.650^c^
Physical activity (METs)	773.7 ± 198.3	760.6 ± 201.21	0.830
Metformin use, n (%)	48 (80)	47 (78)	0.499
Glibenclamide use, n (%)	33 (53)	31 (49)	0.580

METs: Metabolic equivalents (MET-minutes/week); MD: Mean/Median of difference

^a^Values are expressed as mean ± SD

^b^Values are expressed as frequency (%)

^c^Chi-square test

^d^Independent samples t-test

Data on dietary intakes (macronutrient and dietary fiber, and some micronutrients including magnesium and zinc) of the participants are presented in Table 2. We realized a non-significant alteration in group mean energy intake throughout the trial (decrease of − 231.71 and − 213.40 kcal in Taurine and placebo group, *P* = 0.093, *p* = 0.098, respectively). Intake of macronutrients decreased in both groups; however, this was not statistically significant (*P* > 0.050).

**Table 2:** Changes in calorie, macronutrients and micronutrients, and dietary fiber throughout the study

Variable	Taurine group (n = 60)	Placebo group (n = 60)	MD (95% CI) *P*-value
Energy (Kcal/d)			
Baseline	1898.33 ± 207.27	1934.88 ± 287.93	36 (−436.1 to 300.8), 0.856^**^
End	1667.06 ± 200.58)	1721.60 ± 293.34)	54 (−145.1 to 35.68), 0.237^***^
MD (95% CI), P^*^	−231.71 (−567.9 to −18.6), 0.093	−213.40 (−447.40 to 15.9), 0.098	
Carbohydrates (g)			
Baseline	324.43 ± 45.94	323.69 ± 46.40	0.80 (−115.25, 99.78), 0.933^**^
End	262.02 ± 55.31	282.68 ± 55.22	20.92 (−57.31, 10.36), 0.187^***^
MD (95% CI), P^*^	−66.39 (−154.1, 21.6), 0.154	−41.25 (−113.34, 31.12), 0.672	
Protein (g)			
Baseline	62.90 ± 21.25	63.79 ± 20.46	−0.89 (−8.21, 6.33), 0.816^**^
End	69.21 ± 18.33	68.52 ± 18.21	0.68 (−5.3, 7.14), 0.836^***^
MD (95% CI), P^*^	6.32 (2.44, 10.21), **0.002**	4.71 (1.21, 7.72), **0.013**	
Fat (g)			
Baseline	64.72 ± 23.01	66.80 ± 21.49	−2.01 (−10.03, 6.59), 0.613^**^
End	67.18 ± 24.03	70.30 ± 26.02	3.12 (−12.23, 5.20), 0.501^***^
MD (95% CI), P^*^	2.45 (−4.1, 8.33), 0.471	3.50 (−3.12, 10.31), 0.342	
Dietary fiber (g)			
Baseline	18.91 ± 7.70	19.83 ± 7.09	−0.91 (−3.2, 2.12), 0.503^**^
End	20.37 ± 9.85	20.87 ± 9.89	−0.49 (−4.12, 3.35), 0.786^***^
MD (95% CI), P^*^	1.45 (−1.06, 2.34), 0.291	1.04 (−1.06, 3.41), 0.425	
Mg (mg/d)			
Baseline	217.22 ± 75.06	224.32 ± 68.53	−7.01 (−39.03, 18.59), 0.592^**^
End	221.40 ± 56.50	220.54 ± 55.12	0.86 (−16.23, 12.20), 0.933^***^
MD (95% CI), P^*^	4.08 (−14.21, 23.33), 0.654	−3.77 (−20.12, 12.31), 0.653	
Zinc (mg/d)			
Baseline	7.62 ± 2.47	7.80 ± 2.31	−0.18 (−1.2, 3.12), 0.889^**^
End	9.77 ± 3.05	7.72 ± 2.51	−2.01 (−2.12, 6.35), 0.342^***^
MD (95% CI), P^*^	2.15 (−1.06, 2.34), 0.298	0.08 (−0.98, 1.41), 0.868	

Significant results are shown in bold type (* P* < 0.05)

Mean (SD) and Mean difference (95% CI) are presented for data

*P based on Paired samples t-test

**P based on Independent samples t-test

***P based on ANCOVA adjusted for baseline values

After an 8-week period of Taurine supplementation, changes in insulin, and HOMA-IR were found to be significant between the two groups. However, no significant changes were observed in other metabolic parameters (FBG, HbA1c, total cholesterol, HDL-C, LDL-C, DBP, and SBP). Generally, a significant decline in serum insulin (− 3.96 ± 1.63 vs. − 1.45 ± 0.8 mmol/L, *P* = 0.048), and HOMA-IR (− 1.27 ± 0.83 vs. − 0.90 ± 0.76 mmol/L, *P* = 0.022) were noticed following the supplementation with Taurine, compared to the placebo. Therefore, Taurine supplementation results in a significant reduction in insulin resistance in comparison to the placebo (Table 3).

**Table 3:** Metabolic parameters of participants before and after treatment

Variable	Taurine group (n = 60)	Placebo group (n = 60)	MD (95% CI) *P*-value
FBS (mg/dl)			
Baseline	148.33 ± 53. 5	150.27 ± 48. 3	1.93 (−22.4 to 17.7), 0.967^**^
End	123.81 ± 54.22	142.43 ± 45.07	−18.5 (−44.6 to 25.6), 0.583^***^
MD (95% CI), P^*^	−24.3 (−51.4, 2.6) 0.065	−7.83 (−23.31, 11.59) 0.431	
HbA1c (%)			
Baseline	8. 4 ± 3.07	8.01 ± 2.43	−0.38 (−1.4 to 1.6), 0.973^**^
End	7.36 ± 2.53	7.83 ± 1.88	−0.55 (−1.8 to 1.25), 0.155^***^
MD (95% CI), P^*^	−1.04 (−3.25, 0.48) 0.316	−0.17 (−1.58, 1.56) 0.802	
Insulin (mU/mL)			
Baseline	14.32 ± 5.50	14.81 ± 4.32	−0.78 (−3.1, 2.4), 0.767^**^
End	10.36 ± 4.31	13.35 ± 5.05	−2.98 (−5.4, −0.3), 0.048^***^
MD (95% CI), P^*^	−3.96 (−7.74, −0.98), **0.019**	−1.45 (−4.6, 1.7), 0.336	
HOMA-IR Index			
Baseline	4.26 ± 1.8	4.73 ± 1.9	−0.49 (−1.8, 0.14), 0.849^**^
End	2.99 ± 1.93	3.82 ± 2.10	−1.17 (−1.85, −0.15), 0.022^***^
MD (95% CI), P^*^	−1.27 (−2.21, −0.85), **0.013**	−0.90 (−2.2, 0.3), 0.133	
SBP (mmHg)			
Baseline	124.02 ± 18.46	125.22 ± 18.42	−1.19 (−7.1, 5.9), 0.725^**^
End	117.12 ± 23.22	121.97 ± 27.51	−17.4 (−10.27, 4.55), 0.304 ^***^
MD (95% CI), P^*^	−6.8 (−13.6, 0.44), 0.051	−3.25 (−9.2, 3.3), 0.326	
DBP (mmHg)			
Baseline	81.26 ± 12.94	82.38 ± 14.85	4.53 (−6.1, 3.1), 0.662^**^
End	78.29 ± 11.20	82.00 ± 17.64	−3.7 (−7.27, 3.3), 0.178^***^
MD (95% CI), P^*^	−2.96 (−5.2, −1.01), **0.013**	−0.38 (−3.2, 2.4), 0.791	
TC (mg/dl)			
Baseline	207.97 ± 23.44	200.92 ± 23.43	7.04 (−30.1, 20.9), 0.872^**^
End	170.98 ± 28.44	185.12 ± 29.97	−14.4 (−34.27, 24.4), 0.475^***^
MD (95% CI), P^*^	−36.21 (−80.6, 16.55), 0.169	−15.1 (−45.1, 13.8), 0.429	
TG (mg/dl)			
Baseline	198.44 ± 26.88)	199.88 ± 24.67	−1.53 (−9.1, 8.7), 0.975^**^
End	164.46 ± 26.27	188.25 ± 28.33	−23.4 (−77.2, 3.5), 0.469 ^***^
MD (95% CI), P^*^	−33.21 (−63.6, −24.5), **0.001**	−11.2 (−37.2, 4.5), 0.237	
HDL (mg/dl)			
Baseline	43.86 ± 38.16	42.98 ± 6.72	0.88 (−2.1, 1.9), 0.987^**^
End	45.86 ± 7.19	43.83 ± 8.87	2.03 (−4.27, 5.55), 0.166^***^
MD (95% CI), P^*^	2.01 (−0.5, 3.44), 0.071	0.88 (−0.7, 4.4), 0.157	
LDL (mg/dl)			
Baseline	124.42 ± 25.10	130.22 ± 21.86	−5.7 (−12.1, 17.9), 0.920^**^
End	100.76 ± 28.43	117.45 ± 25.75	−17.4 (−34.27, −0.55), 0.722^***^
MD (95% CI), P^*^	−23.21 6.1, 40.1), **0.008**	−12.2 (−25, 3.9), 0.125	

Significant results are shown in bold type (* P* < 0.05)

Mean (SD) and Mean difference (95% CI) are presented for data. MD: mean difference; FBS: Fasting blood sugar; HbA1c: glycosylated hemoglobin; HOMA-IR: homeostasis model of assessment-estimated insulin resistance; SBP: Systolic Blood Pressure; DBP: Diastolic Blood Pressure

*p* values indicate comparison within groups based on paired t test

*P based on Paired samples t-test

** *P* based on Independent samples t-test

*** *P* based on ANCOVA adjusted for baseline values, weight, and calorie intake

A remarkable elevation of serum TAC concentration (65.7 ± 88.4 vs. 16.54 ± 64.7 mmol/L, *P* = 0.015), and decrease in hs-CRP levels (− 1.29 ± 0.70 vs. − 0.64 + 1.27 mg/dL, *P* = 0.001), TNF levels (− 18.74 ± 10.33 vs. − 5.07 + 1.27 ng/ml, *P* = 0.002), and MDA levels (− 41.7 ± 63.73 vs. − 10.29 + 67.6 nmol/mL, *P* = 0.033) were detected following supplementation with Taurine compared with the placebo. Therefore, Taurine led to a significant reduction in biomarkers of oxidative stress and meta-inflammation in comparison to the placebo (Table 4).

**Table 4:** Effect of taurine supplementation on oxidative stress and inflammatory markers

Variable	Taurine group (n = 60)	Placebo group (n = 60)	MD (95% CI), P-value
TAC (mmol/L)			
Before	126.85 ± 39.82	131.36 ± 38.83	4.5 (−18.86 to 8.79), 0.531^a^
After	192.61 ± 91.77	147.52 ± 107.12	45 (9. 6 to 81.1), **0.015**^b^
MD (95% CI), *P*-value^c^	65.73 (41.7 to 88.8), **0.001**	16.54 (−11.7 to 44.7), 0.256	
MDA (nmol/mL)			
Before	170.79 ± 61.47	183.38 ± 125.08	−12.20 (–48.7 to 12.33), 0.485^a^
After	129.61 ± 109.60	172.46 ± 128.19	−43.6 (−83.7 to −3.57), **0.033**^b^
MD (95% CI), *P*-value^c^	−41.70 (−69.6 to −11.74), **0.007**	−10.29 (−32.9 to 24.4), 0.614	
HsCRP (mg/dL)			
Before	3.17 ± 1.88	3.01 ± 1.79	0.41 (−0.55 to 0.85), 0.642^a^
After	1.87 ± 2.08	3.66 ± 3.17	1.76 (−2.7 to −0.88), **0.001**^b^
MD (95% CI), *P*-value^c^	−1.29 (−2.6 to −0.54), **0.001**	−0.64 (−0.50 to 1.25), 0.157	
TNF-alpha (ng/ml)			
Before	33.18 ± 25.27	30.90 ± 24.53	2.28 (−6.66 to 10.45), 0.616^a^
After	14.42 ± 10.50	25.82 ± 26.27	11.01 (−18.3 to −4.3), **0.002**^b^
MD (95% CI), *P*-value^c^	−18.74 (−20.6 to −11.87), **0.001**	−5.07 (−14.50 to 420), 0.291	

Significant results are shown in bold type (* P* < 0.05)

Hs-CRP: high-sensitivity C-reactive protein, MDA: malondialdehyde, TAC: total antioxidant capacity

Values are expressed as mean (SD)

^a^Independent samples t-test

^b^Adjusted for baseline values, weight, and calorie intake using the analysis of covariance (ANCOVA) test

^c^Paired-samples t-test

Table 5 shows data of pre- and post-intervention biomarkers of endothelial markers in both groups. A significant decrease in VCAM concentration (− 1.12 ± 0.77, vs. − 0.14 ± 0.22 mmol/L, *P* = 0.001), ICAM levels (− 12.1 ± 3.70 vs. − 2.49 + 0.98 mg/L, *P* = 0.029), E-Selectin levels (− 10.94 ± 2.89 vs. − 1.51 + 0.74 mg/L, *P* = 0.028) and MMP-9 levels (− 53.18 ± 11.70 vs. − 19.1 + 7.98 mg/L, *P* = 0.049) were identified resulting the Taurine supplementation compared placebo group, respectively. Taurine supplementation resulted in a significant decrease in biomarkers of endothelial dysfunction as compared to placebo group.

**Table 5:** The Effects of taurine supplementation on biomarkers of endothelial markers

Variable	Taurine group (n = 60)	Placebo group (n = 60)	MD (95% CI), *P*-value
VCAM (µg.ml)			
Before	3.14 ± 0.94	3.82 ± 1.25	0.68 (−1.86 to 0.45), 0.230^a^
After	2.10 ± 0.08	3.68 ± 1.15	−1.67 (−2. 7 to −0.7), **0.001**^b^
MD (95% CI), *P*-value^c^	−1.12 (−1.81 to −0.43), **0.002**	−0.14 (−0.4 to 0.13), 0.321	
ICAM (ng/mL)			
Before	81.22 ± 11.64	79.15 ± 9.84	2.2 (–1.7 to 5.33), 0.294^a^
After	68.5 ± 12.25	76.2 ± 14.62	−7.6 (−14.7 to −0.88), **0.029**^b^
MD (95% CI), *P*-value^c^	−12.1 (−18.6 to −6.74), **0.001**	−2.49 (−5.9 to 0.1), 0.054	
E Selectin (mg/L)			
Before	82.42 ± 18.27	79.2 ± 24.53	3.27(−1.66 to 3.77), 0.153^a^
After	71.57 ± 14.50	77.01 ± 26.27	−6.1 (−11.3 to −0.68), **0.028**^b^
MD (95% CI), *P*-value^c^	−10.94 (−17.6 to −2.87), **0.002**	−1.51 (−5.50 to 0.20), 0.081	
MMP-9 (ng.ml.)			
Before	193.65 ± 55.99	179 ± 495.46	14.6(−7.2 to 38.45), 0.193^a^
After	140.23 ± 49.82	160.55 ± 385.85	−20.1 (−38.3 to −0.98), **0.049**
MD (95% CI), *P*-value^c^	−53.18 (−0.8 to −28.17), **0.001**	−19.1 (−1.50 to 0.20), 0.053	

Significant results are shown in bold type (* P* < 0.05)

VCAM: vascular cell adhesion molecule, ICAM: Intercellular Adhesion Molecule 1, MMP-9: Matrix metallopeptidases

Values are expressed as mean (SD)

^a^Independent samples t-test

^b^Adjusted for baseline values and, duration of diabetes, and energy intake changes using the analysis of covariance (ANCOVA) test

^c^Paired-samples t-test

## Discussion

This study’s aim was to evaluate the effect of Taurine on endothelial dysfunction markers, oxidative stress, inflammation, and glycemic control in type 2 diabetic subjects (T2DM). The results of our study for the first time showed that Taurine improved endothelial function indicators including reduction in the ICAM, VCAM, and MMP-9 levels. Furthermore, Taurine had noteworthy effects on some CV risk factors including BP, glycemic control, and inflammation, and oxidative stress markers. Moreover, participants who lost at least of 2.5 kg in weight post-intervention had considerably enhanced cardiometabolic risk assessment compared to those with unremarkable weight loss (data not shown). When results were stratified by weight changes 25% of the participants experienced weight losses more than 2.5 kg by the end of the study (including 17 subjects of the Taurine group and 13 subjects of the placebo group), but regardless of the given supplement, patients losing > 2.5 kg had a greater decrease in CV risk factors in comparison to those who lost < 2.5 kg (data not shown).

Our results demonstrated that an eight-week period of Taurine supplementation enhanced the serum insulin, and HOMA-IR in the Taurine group; although, the levels of FBG and HbA1c did not significantly differ between the groups in study. A similar conclusion was reached by previous surveys in diabetic experimental animal models [30, 31]. A similar pattern of results was obtained in a pilot clinical trial in which the effect of 3 g/day Taurine supplementation was studied [17]. Contrary to our findings, Shari et al. [32] did not find the effect of 1000 mg Taurine for 12 weeks’ on glycemic control in patients with T2DM. It has been assumed that the conflicting findings reported in the literature can be largely attributed to differences in the dose of supplementation, range of glycemic levels, and duration of intervention. The main mechanisms by which Taurine supplementation might improve insulin and HOMA-IR are not well understood. However, Taurine may directly by activate AMP-activated protein kinas) AMPK( in skeletal muscles, or pancreatic islets cells [19, 20]. Another possible mechanism is preventing of the hepatic glucose synthesis in different ways including phosphorylation of the insulin receptor Β-subunit)IRβ(, the reduced glucagon activity in the liver and the increased levels of the uncoupling protein 1 (UCP1) in adipose tissue [19]. In addition to the anti-diabetic effect by regulating activity of the pancreatic cells, the glucose lowering and anti-inflammatory effects of Taurine are further effects of this amino acids on glycemic control [17, 21, 30]. However, in this study, there was not a significant fall in FBG or HbA1c levels in the intervention group, likely due to the inadequate period of treatment (8 weeks). Since erythrocytes have a long-life span, (120 days), a longer duration of treatment is required to observe the possible changes in HbA1c.

Our findings showed that inflammatory and oxidative stress markers (hs-CRP, TNF, TAC, and MDA) were reduced by Taurine. These findings support the idea that Taurine play a significant anti-inflammatory part. Also, our finding is consistent with previous studies showing the protective functions of Taurine against oxidative stress and inflammation [33, 34]; Silva et al*.* [35] revealed that Taurine improve oxidative stress in skeletal muscles. A similar conclusion was drawn by Ahmadian et al*.* [33] which reported that Taurine has anti-inflammatory and cytoprotective effects in patients with heart failure. However, some reports showed that supplementation with 3 g/d of Taurine for 16 weeks did not reduce oxidative stress among patients with T2DM [36]; this can largely be ascribed to the higher inflammatory markers as a result of poor glycemic control among patients with T2DM.

It has been assumed that the anti-inflammatory properties of Taurine arise from its antioxidant capacity to offset hypochlorous acid by the formation of Taurine chloramine [6, 10, 11]. Accordingly, the production of Taurine chloramine at the site of inflammation can regulate the synthesis and secretion of proinflammatory cytokines including TNF, IL-6, and IL-8. Furthermore, Taurine halts generation of superoxides in mitochondria [37].

Patients with T2DM are susceptible to experience numerous challenges including abnormalities in lipid profiles. Another result of the present trial is that Taurine supplementation in patients with T2DM for 8 weeks did not significantly affect lipid profiles. In other studies Taurine through increasing the cholesterol conversion into bile acids, up-regulation of LDL receptors, as well as decreasing the hepatic cholesterol ester pool was shown to improved lipid panel by [38, 39].

Endothelial dysfunction is an early event in development of atherosclerosis and subsequent CVD events that is frequently seen in patients with T2DM [2, 7, 9]. As the main finding, this study showed that in patients with T2DM Taurine exposure for 8 weeks significantly decreased the biomarkers related to endothelial dysfunction including VCAM, ICAM-1, and MMP-9. Although A few studies have considered the effects of Taurine on endothelial markers, to the best of our knowledge, this study was the first clinical trial reporting the effects of Taurine on the biomarkers related to endothelial dysfunction in T2DM. This finding was aligned with those reported by Fennessy, et al*.* showing that Taurine supplementation improved endothelial function [40]. Similar results were demonstrated by an experimental animal model whereby Taurine was found to reduce acute hyperglycemia-induced endothelial markers in male Sprague Dawley rats [22]. Similarly, Taurine restored endothelial function in type I diabetic rats [41]. There are some mechanisms underlaying the endothelial-protective roles of Taurine including lowering vascular NADPH, restoration of phosphorylation of endothelial NOS (eNOS), and enhancing expression of extracellular superoxide dismutase (EcSOD) [42]. Also, a previous survey confirmed that Taurine by scavenging ROS and attenuating lipid peroxidation has a remarkable antioxidant activity [42].

Interruption of the ROS activity, scavenging ROS, and regeneration of thiol groups are probably the most likely mechanisms underlying the beneficial effect of Taurine in diabetic patients [10, 33]. Furthermore, a pile of studies showed a reverse correlation between plasma concentrations of taurine and FBS as well as diabetes complications [14]. As a functional nutrient, taurine plays a significant part in detoxification, osmoregulation, calcium homeostasis, neuromodulator, and cytoprotection. Also, taurine can alter insulin signaling pathway and regulate the *β*-cell insulin secretion ability, leading to the more efficient control of glucose metabolism [12, 25, 40]. In conclusion, based on the European Food Safety Authority (EFSA) report in 2012, the daily application of taurine up to 3000 mg seems to be safe, as we showed the same finding in the present study. Although, there were not significant adverse effects in our study, some studies reported a few adverse effects including nausea, vomiting, headache, stomach pain and rarely inhibition of cytochrome P450 enzyme [43, 44]. It’s unclear whether these effects are directly related to the taurine or they may arise from other impurities. Thus, it seems crucial to assess both the concentrations of taurine in the blood and the adverse effects of taurine at the relevant doses in the future studies.

The current study has some limitations, including not assessing the brachial artery flow‑mediated dilation (FMD), as well as the short duration of the supplementations. Long-standing supplementation would be required to show whether Taurine supplementation positively regulates glycemic control. Aside from that, this supplementation could be beneficial for larger confirmatory studies.

## Conclusion

Overall, we found a positive effect of Taurine supplementation on endothelial markers, as well as metabolic and inflammatory biomarkers, in patients with T2DM. However, anthropometric, blood pressure and lipid profiles did not vary significantly during the study. Considering these results, and the fact that Taurine is available and is low-cost, Taurine supplementation may be a useful therapy or co-therapy in prevention of endothelial dysfunction in patients with T2DM.

## Data Availability

All data generated and analyzed during this study are included in the manuscript.

## Abbreviations

AGEs: Advanced glycation end products
AMPK: AMP-activated protein kinase
FBS: Fasting blood sugar
HDL-C: High-density lipoprotein- cholesterol
HOMA-IR: Homeostatic model assessment-insulin resistance
IRS: Insulin receptor substrate
LDL-C: Low-density lipoprotein-cholesterol
PGC: PPARγ coactivator
ROS: Reactive oxygen species
TC: Total cholesterol
T2DM: Type 2 diabetes mellitus
TG: Triglyceride
VLDL: Very low-density lipoprotein-cholesterol
