# Correction to: Protective and therapeutic effectiveness of taurine supplementation plus low calorie diet on metabolic parameters and endothelial markers in patients with diabetes mellitus: a randomized, clinical trial

**DOI:** 10.1186/s12986-022-00697-x

**Published:** 2022-09-09

**Authors:** Jalal Moludi, Shaimaa A. Qaisar, Mustafa M. Kadhim, Yasin Ahmadi, Mina Davari

**Affiliations:** 1 School of Nutritional Sciences and Food Technology, Kermanshah University of Medical Sciences, Kermanshah, 5166614711 Iran; 2 Behavioral Disease Research Center, Kermanshah University of Medical Sciences, Kermanshah, Iran; 3 Chemistry Department, College of Education, University of Garmian, Sulimmania, Iraq; 4 Department of Medical Laboratory Techniques, Dijlah University College, Baghdad, 10021 Iraq; 5 Medical Laboratory Techniques Department, Al-Farahidi University, Baghdad, Iraq; 6 Tabriz University of Medical Sciences, Tabriz, Iran

## Correction to: Nutrition & Metabolism (2022) 19:49 10.1186/s12986-022-00684-2

Following publication of the original article [1], the authors identified an error in affiliation 6.

The incorrect affiliation is: Tabriz University of Medical Sciences, Kermanshah, Iran.

The correct affiliation is: Tabriz University of Medical Sciences, Tabriz, Iran.

The original article [1] has been corrected.
